# Supplementary material for: Temporal pattern and synergy influence activity of ERK signaling pathways during L-LTP induction
Source: eLife. 2021 Aug 10;10:e64644. doi: 10.7554/eLife.64644 (PMC8363267; doi:10.7554/eLife.64644)
Supplement: Figure 1—source data 1. [file elife-64644-fig1-data1.docx]

**Figure 1 –** **Source Data 1:** Reactions and rates constant involved in signaling pathway leading to Ras and Rap1 activation

| Reaction equation | K_f_ (nM^-1^ Sec^-1^) | | K_b_ (Sec^-1^) | K_cat_ (Sec^-1^) | Reference |
| --- | --- | --- | --- | --- | --- |
| *Gby + Src* $\boldsymbol{\leftrightarrow}$ *Src_Gby* | | 2.00E-04 | 1.00E-01 |  | Estimated |
| *Grb2 + Sos* $\boldsymbol{\leftrightarrow}$ *Grb2_Sos* | | 2.50E-04 | 1.68E-02 |  | Chook et al., 1996; Jain and Bhalla, 2014 |
| *Src_Gby +Shc* $\boldsymbol{\leftrightarrow}$ *pShc +Src_Gbg* | | 8.00E-03 | 1.28E-01 | 3.20E-02 | Estimated |
| *pShc + Src_Grb2_Sos* $\boldsymbol{\leftrightarrow}$ *pShc_Grb2_Sos* | | 5.00E+00 | 1.00E-01 |  | Bhalla and Iyengar, 1999; Jain and Bhalla, 2014; Sasagawa et al., 2005 |
| *pShc_Grb2_Sos + RasGDP* $\boldsymbol{\leftrightarrow}$ *pShc_Grb2_Sos + RasGTP* | | 1.98E-02 | 8.00E-01 | 2.00E-01 | Bhalla and Iyengar, 1999; Jain and Bhalla, 2014; Sasagawa et al., 2005 |
| *pShc* $\boldsymbol{\leftrightarrow}$ *Shc* | | 2.00E-01 | 0.00E+00 |  | Jain and Bhalla, 2014 |
| *ppERK +pShc_Grb2_Sos*$\boldsymbol{\leftrightarrow}$*ppERK +pShc_Grb2 + pSos* | | 1.95E-02 | 4.00E+01 | 1.00E+01 | Jain and Bhalla, 2014; Sasagawa et al., 2005 |
| *pSos*$\boldsymbol{\leftrightarrow S}$*os* | | 1.00E+00 |  |  | Jain and Bhalla, 2014 |
| *CaMCa4 + Ras-GRF2* $\boldsymbol{\leftrightarrow}$ *Ras-GRF2_CaMCa4* | | 6.00E-02 | 9.24E-01 |  | Jin et al., 2014 |
| *Ras-GRF2_CaMCa4 + RasGDP* $\boldsymbol{\leftrightarrow}$ *Ras-GRF2_CaMCa4 + RasGTP* | | 1.98E-02 | 8.00E-01 | 2.00E-01 | Farnsworth et al., 1995; Jin et al., 2014, |
| *PKAc + Src* $\boldsymbol{\leftrightarrow}$ *pSrc + PKAc* | | 2.04E+00 | 8.00E+01 | 2.00E+01 | Jain and Bhalla, 2014 |
| *pSrc + Cbl* $\boldsymbol{\leftrightarrow}$ *pCbl + Src* | | 4.00E-01 | 1.60E+02 | 4.00E+01 | Jain and Bhalla, 2014 |
| *Crk_C3G + pCbl* $\boldsymbol{\leftrightarrow}$*Crk_C3G_pCbl* | | 1.00E-03 | 2.00E-01 |  | Jain and Bhalla, 2014; Knudsen et al., 1994; Sasagawa et al., 2005 |
| *pCbl* $\boldsymbol{\leftrightarrow}$*Cbl* | | 1.00E+02 |  |  | Jain and Bhalla, 2014 |
| *pSrc* $\boldsymbol{\leftrightarrow}$*Src* | | 1.00E+02 |  |  | Jain and Bhalla, 2014 |
| *Crk_C3G_pCbl + Rap1GDP* $\boldsymbol{\leftrightarrow}$*Crk_C3G_pCbl + Rap1GTP* | | 1.01E-02 | 8.00E-02 | 2.00E-02 | Gotoh et al., 1995; Jain and Bhalla, 2014; Sasagawa et al., 2005 |
| *Epac + cAMP* $\boldsymbol{\leftrightarrow}$ *Epac_cAMP* | | 1.21E-04 | 1.45E-01 |  | de Rooij et al., 2000; Luczak et al., 2017 |
| *Epac_cAMP + Rap1GDP* $\boldsymbol{\leftrightarrow}$ *Epac_cAMP + Rap1GTP* | | 1.20E-03 | 9.60E-01 | 2.40E-01 | Estimated |
| *Rap1GTP+Rap1Gap* $\boldsymbol{\leftrightarrow}$ *Rap1GDP* | | 2.00E-01 | 2.00E+02 |  | Daumke et al., 2004; Jain and Bhalla, 2014; Sasagawa et al., 2005 |
| *RasGTP+ RasGap* $\boldsymbol{\leftrightarrow}$ *RasGDP* | | 4.95E-02 | 4.00E+01 |  | Bhalla and Iyengar, 1999; Jain and Bhalla, 2014 |

**REFERENCES**

1. Bhalla, U.S., Iyengar, R., 1999. Emergent Properties of Networks of Biological Signaling Pathways. Science 283, 381–387.
2. Chook, Y.M., Gish, G.D., Kay, C.M., Pai, E.F., Pawson, T., 1996. The Grb2-mSos1 Complex Binds Phosphopeptides with Higher Affinity than Grb2*. Journal of Biological Chemistry 271, 30472–30478.
3. Daumke, O., Weyand, M., Chakrabarti, P.P., Vetter, I.R., Wittinghofer, A., 2004. The GTPase-activating protein Rap1GAP uses a catalytic asparagine. Nature 429, 197–201.
4. de Rooij, J., Rehmann, H., van Triest, M., Cool, R.H., Wittinghofer, A., Bos, J.L., 2000. Mechanism of Regulation of the Epac Family of cAMP-dependent RapGEFs. J. Biol. Chem. 275, 20829–20836.
5. Farnsworth, C.L., Freshney, N.W., Rosen, L.B., Ghosh, A., Greenberg, M.E., Feig, L.A., 1995. Calcium activation of Ras mediated by neuronal exchange factor Ras-GRF. Nature 376, 524–7.
6. Gotoh, T., Hattori, S., Nakamura, S., Kitayama, H., Noda, M., Takai, Y., Kaibuchi, K., Matsui, H., Hatase, O., Takahashi, H., Kurata, T., Matsuda, M., 1995. Identification of Rap 1 as a target for the Crk SH3 domain-binding guanine nucleotide-releasing factor C3G. Molecular and cellular biology 15, 6746–6753.
7. Jain, P., Bhalla, U.S., 2014. Transcription Control Pathways Decode Patterned Synaptic Inputs into Diverse mRNA Expression Profiles. PLoS ONE 9, e95154.
8. Jin, S.-X., Bartolome, C., Arai, J.A., Hoffman, L., Uzturk, B.G., Kumar-Singh, R., Waxham, M.N., Feig, L.A., 2014. Domain Contributions to Signaling Specificity Differences Between Ras-Guanine Nucleotide Releasing Factor (Ras-GRF) 1 and Ras-GRF2. Journal of Biological Chemistry 289, 16551–16564.
9. Knudsen, B.S., Feller, S.M., Hanafusa, H., 1994. Four proline-rich sequences of the guanine-nucleotide exchange factor C3G bind with unique specificity to the first Src homology 3 domain of Crk. J Biol Chem 269, 32781–32787.
10. Luczak, V., Blackwell, K.T., Abel, T., Girault, J.-A., Gervasi, N., 2017. Dendritic diameter influences the rate and magnitude of hippocampal cAMP and PKA transients during β-adrenergic receptor activation. Neurobiol Learn Mem 138, 10–20.
11. Sasagawa, S., Ozaki, Y., Fujita, K., Kuroda, S., 2005. Prediction and validation of the distinct dynamics of transient and sustained ERK activation. Nature Cell Biology 7, 365–373.
