# Supplementary material for: Temporal pattern and synergy influence activity of ERK signaling pathways during L-LTP induction
Source: eLife. 2021 Aug 10;10:e64644. doi: 10.7554/eLife.64644 (PMC8363267; doi:10.7554/eLife.64644)
Supplement: Figure 1—source data 2. [file elife-64644-fig1-data2.docx]

**Figure 1 –** **Source Data 2:** Reaction and rates constant involved in signaling pathway of SynGap. Rates were optimized from Oh et al., 2004 and Walkup IV et al., 2015 (Miningou and Blackwell, 2018).

| Reaction equation | K_f_ (nM^-1^ Sec^-1^) | K_b_ (Sec^-1^) | K_cat_ (Sec^-1^) | Reference |
| --- | --- | --- | --- | --- |
| *CKpCaMCa4 + SynGAP* $\boldsymbol{\leftrightarrow}$ *pSynGAP + CKpCaMCa4* | 3.07E-01 | 5.87E+01 | 6.48E-01 | Miningou and Blackwell, 2018 |
| *Ras1GTP + SynGAP* $\boldsymbol{\leftrightarrow}$ *Ras1GDP + SynGAP* | 8.20E-04 | 8.20E-01 | 2.05E-01 | Miningou and Blackwell, 2018 |
| *Rap1GTP + SynGAP* $\boldsymbol{\leftrightarrow}$ *Rap1GDP + SynGAP* | 3.20E-03 | 3.20E+00 | 8.00E-01 | Miningou and Blackwell, 2018 |
| *RasGTP + pSynGAP* $\boldsymbol{\leftrightarrow}$ *RasGDP + pSynGAP* | 1.12E-03 | 1.11E+00 | 2.79E-01 | Miningou and Blackwell, 2018 |
| *Rap1GTP + pSynGAP* $\boldsymbol{\leftrightarrow}$ *Rap1GDP + pSynGAP* | 6.14E-03 | 6.14E+00 | 1.53E+00 | Miningou and Blackwell, 2018 |
| *pSynGAP* $\boldsymbol{\leftrightarrow}$ *SynGAP* | 1.00E+00 | 0.00E+00 |  | Estimated |
| *pSynGAP* $\boldsymbol{\leftrightarrow S}$*ynGap_dendrite* | 2.50E-02 | 2.50E-02 |  | Araki et al., 2015 |

**REFERENCES**

1. Araki, Y., Zeng, M., Zhang, M., Huganir, R.L., 2015. Rapid Dispersion of SynGAP from Synaptic Spines Triggers AMPA Receptor Insertion and Spine Enlargement during LTP. Neuron 85, 173–189.
2. Oh, J.S., Manzerra, P., Kennedy, M.B., 2004. Regulation of the Neuron-specific Ras GTPase-activating Protein, synGAP, by Calcium /Calmodulin-dependent Protein Kinase II. Journal of Biological Chemistry 279, 17980–17988.
3. Miningou Zobon N and Blackwell KT, 2018. Github. https://github.com/neurord/neurord_fit/tree/master/syngap_ras. 8f7ae28
4. Walkup IV, W.G., Washburn, L., Sweredoski, M.J., Carlisle, H.J., Graham, R.L., Hess, S., Kennedy, M.B., 2015. Phosphorylation of Synaptic GTPase-activating Protein (synGAP) by Ca2+/Calmodulin-dependent Protein Kinase II (CaMKII) and Cyclin-dependent Kinase 5 (CDK5) Alters the Ratio of Its GAP Activity toward Ras and Rap GTPases. Journal of Biological Chemistry 290, 4908–4927.
