# Supplementary material for: Temporal pattern and synergy influence activity of ERK signaling pathways during L-LTP induction
Source: eLife. 2021 Aug 10;10:e64644. doi: 10.7554/eLife.64644 (PMC8363267; doi:10.7554/eLife.64644)
Supplement: Figure 1—source data 3. [file elife-64644-fig1-data3.docx]

**Figure 1 – Source Data 3:** Reactions and rates constant involved in core ERK signaling pathway

| Reaction equation | K_f_ (nM^-1^ Sec^-1^) | K_b_ (Sec^-1^) | K_cat_ (Sec^-1^) | Reference |
| --- | --- | --- | --- | --- |
| *Rap1GTP + B-Raf* $\boldsymbol{\leftrightarrow}$ *B-Raf_Rap1GTP* | 6.00E-02 | 1.00E+00 |  | Jain and Bhalla, 2014; Sasagawa et al., 2005 |
| *Rap1GTP + Raf-1* $\boldsymbol{\leftrightarrow}$ *RapGTP_Raf-1 + RasGTP* | 6.00E-03 | 5.00E-01 |  | Jain and Bhalla, 2014; Sasagawa et al., 2005 |
| *B-Raf_Rap1GTP + MEK* $\boldsymbol{\leftrightarrow}$ *pMEK + B-Raf_Rap1GTP* | 9.38E-03 | 1.20E+00 | 3.00E-01 | ain and Bhalla, 2014; Sasagawa et al., 2005 |
| *B-Raf_Rap1GTP + pMEK* $\boldsymbol{\leftrightarrow}$ *ppMEK + B-Raf_Rap1GTP* | 9.38E-03 | 1.20E+00 | 3.00E-01 | Jain and Bhalla, 2014; Sasagawa et al., 2005 |
| *RasGTP + Raf1* $\boldsymbol{\leftrightarrow}$ *Raf1_ RasGTP* | 6.00E-02 | 1.00E+00 |  | Block et al., 1996; Force et al., 1994; Jain and Bhalla, 2014 |
| *2Raf1_ RasGTP* $\boldsymbol{\leftrightarrow}$*dRaf1_ RasGTP* | 1.00E-02 | 5.00E-01 |  | Estimated |
| *RasGTP + bRaf* $\boldsymbol{\leftrightarrow}$ *bRaf_RasGTP* | 6.00E-03 | 5.00E-01 |  | Jain and Bhalla, 2014; Yamamori et al., 1995 |
| *dRaf1_RasGTP + MEK* $\boldsymbol{\leftrightarrow}$ *pMEK + dRaf1_RasGTP* | 1.89E-02 | 2.40E+00 | 6.00E-01 | Estimated |
| *dRaf1_RasGTP + pMEK* $\boldsymbol{\leftrightarrow}$ *ppMEK + dRaf1_RasGTP* | 1.89E-02 | 2.40E+00 | 6.00E-01 | Estimated |
| *B-Raf _RasGTP + MEK* $\boldsymbol{\leftrightarrow}$ *pMEK + B-Raf _RasGTP* | 6.29E-03 | 8.00E-01 | 2.00E-01 | Jain and Bhalla, 2014; VanScyoc et al., 2008 |
| *B-Raf _RasGTP + pMEK* $\boldsymbol{\leftrightarrow}$ *ppMEK + B-Raf _RasGTP* | 6.29E-03 | 8.00E-01 | 2.00E-01 | Jain and Bhalla, 2014; VanScyoc et al., 2008 |
| *ppMEK + PP2A* $\boldsymbol{\leftrightarrow}$ *pMEK + PP2A* | 1.92E-03 | 2.40E+01 | 6.00E+00 | Jain and Bhalla, 2014; Takai and Mieskes, 1991 |
| *ppMEK + PP2A* $\boldsymbol{\leftrightarrow}$ *MEK + PP2A* | 1.92E-03 | 2.40E+01 | 6.00E+00 | Jain and Bhalla, 2014; Takai and Mieskes, 1991 |
| *ppMEK + ERK* $\boldsymbol{\leftrightarrow}$ *pERK + ppMEK* | 3.24E-02 | 1.20E+00 | 3.00E-01 | Haystead et al., 1992; Jain and Bhalla, 2014 |
| *ppMEK + ERK* $\boldsymbol{\leftrightarrow}$ *ppERK + ppMEK* | 3.24E-02 | 1.20E+00 | 3.00E-01 | Haystead et al., 1992; Jain and Bhalla, 2014 |
| *ppERK + MKP-1* $\boldsymbol{\leftrightarrow}$ *pERK+ MKP-1* | 1.50E-01 | 1.60E+01 | 4.00E+00 | Jain and Bhalla, 2014 |
| *pERK + MKP-1* $\boldsymbol{\leftrightarrow}$ *ERK+ MKP-1* | 1.50E-01 | 1.60E+01 | 4.00E+00 | Jain and Bhalla, 2014 |

**REFERENCE**

1. Block, C., Janknecht, R., Herrmann, C., Nassar, N., Wittinghofer, A., 1996. Quantitative structure-activity analysis correlating Ras/Raf interaction in vitro to Raf activation in vivo. Nat Struct Mol Biol 3, 244–251.
2. Force, T., Bonventre, J.V., Heidecker, G., Rapp, U., Avruch, J., Kyriakis, J.M., 1994. Enzymatic characteristics of the c-Raf-1 protein kinase. Proceedings of the National Academy of Sciences 91, 1270–1274.
3. Haystead, T.A.J., Dent, P., Wu, J., Haystead, C.M.M., Sturgill, T.W., 1992. Ordered phosphorylation of p42mapk by MAP kinase kinase. FEBS Letters 306, 17–22.
4. Jain, P., Bhalla, U.S., 2014. Transcription Control Pathways Decode Patterned Synaptic Inputs into Diverse mRNA Expression Profiles. PLoS ONE 9, e95154.
5. Sasagawa, S., Ozaki, Y., Fujita, K., Kuroda, S., 2005. Prediction and validation of the distinct dynamics of transient and sustained ERK activation. Nature Cell Biology 7, 365–373.
6. Takai, A., Mieskes, G., 1991. Inhibitory effect of okadaic acid on the p-nitrophenyl phosphate phosphatase activity of protein phosphatases. Biochemical Journal 275, 233–239.
7. VanScyoc, W.S., Holdgate, G.A., Sullivan, J.E., Ward, W.H.J., 2008. Enzyme Kinetics and Binding Studies on Inhibitors of MEK Protein Kinase. Biochemistry 47, 5017–5027.
8. Yamamori, B., Kuroda, S., Shimizu, K., Fukui, K., Ohtsuka, T., Takai, Y., 1995. Purification of a Ras-dependent mitogen-activated protein kinase kinase kinase from bovine brain cytosol and its identification as a complex of B-Raf and 14-3-3 proteins. J Biol Chem 270, 11723–11726.
