# Supplementary material for: Temporal pattern and synergy influence activity of ERK signaling pathways during L-LTP induction
Source: eLife. 2021 Aug 10;10:e64644. doi: 10.7554/eLife.64644 (PMC8363267; doi:10.7554/eLife.64644)
Supplement: Figure 1—source data 4. [file elife-64644-fig1-data4.docx]

**Figure 1 –** **Source Data 4:** Reaction and rates constant involved in signaling pathways from calcium to CaMKII. Where indicated, CaMKII was optimized using De Koninck and Schulman, 1998 (Blackwell, 2019)

| Reaction equation | K_f_ (nM^-1^ Sec^-1^) | K_b_ (Sec^-1^) | K_cat_ (Sec^-1^) | Reference |
| --- | --- | --- | --- | --- |
| *Ca+ pmca* $\boldsymbol{\leftrightarrow}$ *pmca + Caext* | 5.00E-01 | 7.00E+00 | 3.50E+00 | Jȩdrzejewska-Szmek et al., 2017; Sedova and Blatter, 1999 |
| *Ca+ ncx* $\boldsymbol{\leftrightarrow}$ *ncx + Caext* | 1.68E-02 | 1.12E+01 | 5.60E+00 | Gall et al., 1999; Jȩdrzejewska-Szmek et al., 2017; Lőrincz et al., 2007 |
| *Caext +Leak* $\boldsymbol{\leftrightarrow}$ *Ca + Leak* | 1.50E-03 | 1.10E+00 | 1.10E+00 | Jȩdrzejewska-Szmek et al., 2017 |
| *Ca + Calbin* $\boldsymbol{\leftrightarrow}$ *CalbinCa* | 2.80E-02 | 1.96E+01 |  | Jȩdrzejewska-Szmek et al., 2017; Schmidt et al., 2007 |
| *CB + Ca* $\boldsymbol{\leftrightarrow}$ *CBCa* | 2.00E-02 | 1.00E+03 |  | Matthews et al., 2013; Matthews and Dietrich, 2015 |
| *Ng + CaM* $\boldsymbol{\leftrightarrow}$ *NgCaM* | 2.80E-02 | 3.60E+01 |  | Jȩdrzejewska-Szmek et al., 2017; Kubota et al., 2007 |
| *CaM+2Ca* $\boldsymbol{\leftrightarrow}$ *CaMCa2C* | 6.00E-03 | 9.10E+00 |  | Brown et al., 1997; Jȩdrzejewska-Szmek et al., 2017 |
| *CaMCa2C + 2Ca* $\boldsymbol{\leftrightarrow}$ *CaMCa4* | 1.00E-01 | 1.00E+03 |  | Jȩdrzejewska-Szmek et al., 2017; Putkey et al., 2003 |
| *CaM+2Ca* $\boldsymbol{\leftrightarrow}$ *CaMCa2N* | 1.00E-01 | 1.00E+03 |  | Brown et al., 1997; Jȩdrzejewska-Szmek et al., 2017 |
| *CaMCa2N + 2Ca* $\boldsymbol{\leftrightarrow}$ *CaMCa4* | 6.00E-03 | 9.10E+00 |  | Jȩdrzejewska-Szmek et al., 2017; Putkey et al., 2003 |
| *CaMCa4+CK* $\boldsymbol{\leftrightarrow}$ *CKCaMCa4* | 1.00E-02 | 1.50E+00 |  | Dupont and Goldbeter, 1998; Jȩdrzejewska-Szmek et al., 2017 |
| *2CKCaMCa* $\boldsymbol{\leftrightarrow}$ *CKpCaMCa4+ CKCaMCa4* | 3.83E-07 |  |  | Blackwell, 2019 |
| *3CKCaMCa* $\boldsymbol{\leftrightarrow}$ *CKpCaMCa4+ 2CKCaMCa4* | 3.56E-10 |  |  | Blackwell, 2019 |
| *4CKCaMCa* $\boldsymbol{\leftrightarrow}$ *CKpCaMCa4+ 3CKCaMCa4* | 2.24E-13 |  |  | Blackwell, 2019 |
| *2 CKpCaMCa4 + 2 CKCaMCa4* $\boldsymbol{\leftrightarrow}$ *3 CKpCaMCa4 + 1 CKCaMCa4* | 1.10E-15 |  |  | Blackwell, 2019 |
| *2 CKpCaMCa4 + 2 CKCaMCa4* $\boldsymbol{\leftrightarrow}$ *3 CKpCaMCa4 + 1 CKCaMCa4* | 3.03E-10 |  |  | Blackwell, 2019 |
| *2 CKpCaMCa4 + 2 CKCaMCa4* $\boldsymbol{\leftrightarrow}$ *3 CKpCaMCa4 + 1 CKCaMCa4* | 2.39E-10 |  |  | Blackwell, 2019 |
| *CKpCaMCa4* $\boldsymbol{\leftrightarrow}$ *CKp + CaMCa4* | 8.00E-04 | 1.00E-02 |  | Dupont and Goldbeter, 1998; Jȩdrzejewska-Szmek et al., 2017 |
| *CKp+PP1* $\boldsymbol{\leftrightarrow}$ *CK + PP1* | 4.00E-05 | 3.40E-01 | 8.60E-02 | Blackwell, 2019 |
| *CKpCaMCa4 + PP1* $\boldsymbol{\leftrightarrow}$ *CKCaMCa4 + PP1* | 4.00E-05 | 3.40E-01 | 8.60E-02 | Blackwell, 2019 |
| *Ip35 + PP1* $\boldsymbol{\leftrightarrow}$ *Ip35PP1* | 1.00E-03 | 1.10E-03 |  | Connor et al., 2000; Huang et al., 1999; Jȩdrzejewska-Szmek et al., 2017 |

**REFERENCES**

1. Blackwell KT, 2019. Github. <https://github.com/neurord/neurord_fit/tree/master/camkii>. aa721d3
2. Brown, S.E., Martin, S.R., Bayley, P.M., 1997. Kinetic control of the dissociation pathway of calmodulin-peptide complexes. J Biol Chem 272, 3389–3397.
3. Connor, J.H., Frederick, D., Huang, H., Yang, J., Helps, N.R., Cohen, P.T.W., Nairn, A.C., DePaoli-Roach, A., Tatchell, K., Shenolikar, S., 2000. Cellular Mechanisms Regulating Protein Phosphatase-1: A KEY FUNCTIONAL INTERACTION BETWEEN INHIBITOR-2 AND THE TYPE 1 PROTEIN PHOSPHATASE CATALYTIC SUBUNIT *. Journal of Biological Chemistry 275, 18670–18675. De Koninck, P., Schulman, H., 1998. Sensitivity of CaM Kinase II to the Frequency of Ca2+ Oscillations. Science 279, 227–230.
4. Dupont, G., Goldbeter, A., 1998. CaM kinase II as frequency decoder of Ca2+ oscillations. Bioessays 20, 607–610.
5. Gall, D., Gromada, J., Susa, I., Rorsman, P., Herchuelz, A., Bokvist, K., 1999. Significance of Na/Ca Exchange for Ca2+ Buffering and Electrical Activity in Mouse Pancreatic β-Cells. Biophysical Journal 76, 2018–2028.
6. Huang, H.B., Horiuchi, A., Watanabe, T., Shih, S.R., Tsay, H.J., Li, H.C., Greengard, P., Nairn, A.C., 1999. Characterization of the inhibition of protein phosphatase-1 by DARPP-32 and inhibitor-2. J Biol Chem 274, 7870–7878.
7. Jȩdrzejewska-Szmek, J., Luczak, V., Abel, T., Blackwell, K.T., 2017. β-adrenergic signaling broadly contributes to LTP induction. PLOS Computational Biology 13, e1005657.
8. Kubota, Y., Putkey, J.A., Waxham, M.N., 2007. Neurogranin Controls the Spatiotemporal Pattern of Postsynaptic Ca2+/CaM Signaling. Biophysical Journal 93, 3848–3859.
9. Lőrincz, A., Rózsa, B., Katona, G., Vizi, E.S., Tamás, G., 2007. Differential distribution of NCX1 contributes to spine–dendrite compartmentalization in CA1 pyramidal cells. Proc Natl Acad Sci U S A 104, 1033–1038.
10. Matthews, E.A., Dietrich, D., 2015. Buffer mobility and the regulation of neuronal calcium domains. Front Cell Neurosci 9, 48.
11. Matthews, E.A., Schoch, S., Dietrich, D., 2013. Tuning local calcium availability: cell-type-specific immobile calcium buffer capacity in hippocampal neurons. J Neurosci 33, 14431–14445.
12. Putkey, J.A., Kleerekoper, Q., Gaertner, T.R., Waxham, M.N., 2003. A New Role for IQ Motif Proteins in Regulating Calmodulin Function *. Journal of Biological Chemistry 278, 49667–49670.
13. Schmidt, H., Kunerth, S., Wilms, C., Strotmann, R., Eilers, J., 2007. Spino-dendritic cross-talk in rodent Purkinje neurons mediated by endogenous Ca2+-binding proteins. The Journal of Physiology 581, 619–629.
14. Sedova, M., Blatter, L.A., 1999. Dynamic regulation of [Ca2+]i by plasma membrane Ca(2+)-ATPase and Na+/Ca2+ exchange during capacitative Ca2+ entry in bovine vascular endothelial cells. Cell Calcium 25, 333–343.
