# Supplementary material for: Temporal pattern and synergy influence activity of ERK signaling pathways during L-LTP induction
Source: eLife. 2021 Aug 10;10:e64644. doi: 10.7554/eLife.64644 (PMC8363267; doi:10.7554/eLife.64644)
Supplement: Figure 1—source data 5. [file elife-64644-fig1-data5.docx]

**Figure 1 –** **Source Data 5:** Reactions and rates constant involved in signaling pathways leading from cAMP to PKA

| Reaction equation | K_f_ (nM^-1^Sec^-1^) | K_b_ (Sec^-1^) | K_cat_ (Sec^-1^) | Reference |
| --- | --- | --- | --- | --- |
| *PKA + 2cAMP* $\boldsymbol{\leftrightarrow}$*PKAcAMP2* | 2.16E-04 | 6.00E-02 |  | Herberg et al., 1996; Jȩdrzejewska-Szmek et al., 2017; Ogreid and Døskeland, 1981 |
| *PKAcAMP2+2cAMP*$\boldsymbol{\leftrightarrow}$*PKAcAMP4* | 3.50E-04 | 6.00E-01 |  | Herberg et al., 1996; Jȩdrzejewska-Szmek et al., 2017; Ogreid and Døskeland, 1982 |
| *PKAcAMP4*$\boldsymbol{\leftrightarrow}$ *PKAr + PKAc* | 2.40E-01 | 2.55E-02 |  | Jȩdrzejewska-Szmek et al., 2017; Zawadzki and Taylor, 2004 |
| *PDE2+cAMP* $\boldsymbol{\leftrightarrow}$ *PDE2cAMP* | 2.00E-05 | 5.00E-01 |  | Blackwell et al., 2019 |
| *PDE2cAMP+cAMP* $\boldsymbol{\leftrightarrow}$*PDE2cAMP2 +AMP* | 5.90E-03 | 5.00E-01 | 5.40E+00 | Blackwell et al., 2019 |
| *PDE4+cAMP* $\boldsymbol{\leftrightarrow}$ *PDE4 +AMP* | 2.16E-02 | 6.90E+01 | 1.72E+01 | Herman et al., 2000; Jȩdrzejewska-Szmek et al., 2017 |

**REFERENCES**

1. Blackwell, K.T., Salinas, A.G., Tewatia, P., English, B., Kotaleski, J.H., Lovinger, D.M., 2019. Molecular mechanisms underlying striatal synaptic plasticity: relevance to chronic alcohol consumption and seeking. European Journal of Neuroscience 49, 768–783.
2. Herberg, F.W., Taylor, S.S., Dostmann, W.R.G., 1996. Active Site Mutations Define the Pathway for the Cooperative Activation of cAMP-Dependent Protein Kinase. Biochemistry 35, 2934–2942.
3. Herman, S.B., Juilfs, D.M., Fauman, E.B., Juneau, P., Menetski, J.P., 2000. Analysis of a mutation in phosphodiesterase type 4 that alters both inhibitor activity and nucleotide selectivity. Mol Pharmacol 57, 991–999.
4. Jȩdrzejewska-Szmek, J., Luczak, V., Abel, T., Blackwell, K.T., 2017. β-adrenergic signaling broadly contributes to LTP induction. PLOS Computational Biology 13, e1005657.
5. Ogreid, D., Døskeland, S.O., 1982. Activation of protein kinase isoenzymes under near physiological conditions. Evidence that both types (A and B) of cAMP binding sites are involved in the activation of protein kinase by cAMP and 8-N3-cAMP. FEBS Lett 150, 161–166.
6. Ogreid, D., Døskeland, S.O., 1981. The kinetics of association of cyclic AMP to the two types of binding sites associated with protein kinase II from bovine myocardium. FEBS Lett 129, 287–292.
7. Zawadzki, K.M., Taylor, S.S., 2004. cAMP-dependent Protein Kinase Regulatory Subunit Type IIβ. Journal of Biological Chemistry 279, 7029–7036.
