# Supplementary material for: Temporal pattern and synergy influence activity of ERK signaling pathways during L-LTP induction
Source: eLife. 2021 Aug 10;10:e64644. doi: 10.7554/eLife.64644 (PMC8363267; doi:10.7554/eLife.64644)
Supplement: Transparent reporting form [file elife-64644-transrepform.docx]

***eLife’s* transparent reporting form**

We encourage authors to provide detailed information *within their submission* to facilitate the interpretation and replication of experiments. Authors can upload supporting documentation to indicate the use of appropriate reporting guidelines for health-related research (see [EQUATOR Network](http://www.equator-network.org/%20)), life science research (see the [BioSharing Information Resource](https://biosharing.org/)), or the [ARRIVE guidelines](http://www.plosbiology.org/article/info:doi/10.1371/journal.pbio.1000412) for reporting work involving animal research. Where applicable, authors should refer to any relevant reporting standards documents in this form.

If you have any questions, please consult our Journal Policies and/or contact us: [editorial@elifesciences.org](mailto:editorial@elifesciences.org).

**Sample-size estimation**

- You should state whether an appropriate sample size was computed when the study was being designed
- You should state the statistical method of sample size computation and any required assumptions
- If no explicit power analysis was used, you should describe how you decided what sample (replicate) size (number) to use

Please outline where this information can be found within the submission (e.g., sections or figure legends), or explain why this information doesn’t apply to your submission:

The sample size was determined prior to running simulations, and was based on prior computational studies in our lab. This information is provided in Simulation and Analysis section in the methods.

**Replicates**

- You should report how often each experiment was performed
- You should include a definition of biological versus technical replication
- The data obtained should be provided and sufficient information should be provided to indicate the number of independent biological and/or technical replicates
- If you encountered any outliers, you should describe how these were handled
- Criteria for exclusion/inclusion of data should be clearly stated
- High-throughput sequence data should be uploaded before submission, with a private link for reviewers provided (these are available from both GEO and ArrayExpress)

Please outline where this information can be found within the submission (e.g., sections or figure legends), or explain why this information doesn’t apply to your submission:

The replicates are neither technical nor biological replicates. The replicates were obtained by using different random seed, which provided variability in the injected amounts and the reactions that occurred. This information is provided in simulation and analysis in the method section

In addition to using different random seeds, we repeated key simulations with variation in molecule concentrations, analogous to biological replicates. Those results are provided in the robustness section of the results.

Exclusion criteria: No data were excluded, no outliers were encountered.

**Statistical reporting**

- Statistical analysis methods should be described and justified
- Raw data should be presented in figures whenever informative to do so (typically when N per group is less than 10)
- For each experiment, you should identify the statistical tests used, exact values of N, definitions of center, methods of multiple test correction, and dispersion and precision measures (e.g., mean, median, SD, SEM, confidence intervals; and, for the major substantive results, a measure of effect size (e.g., Pearson's r, Cohen's d)
- Report exact p-values wherever possible alongside the summary statistics and 95% confidence intervals. These should be reported for all key questions and not only when the p-value is less than 0.05.

Please outline where this information can be found within the submission (e.g., sections or figure legends), or explain why this information doesn’t apply to your submission:

To test linearity of ppERK in response to changes in duration or concentration (Figures 3 &4), we assessed the adjusted R2 and Akaike information criteria for curve fit to fit a line, a Hill equation, and a logarithmic equation, as explained in the Methods, Simulation and Analysis section. The results for the statistical tests are provided in Figure 3- Supplementary Table 1, and Figure 4- Supplementary Table 1.

To test linear combination of signaling pathways (Figures 3, 5, 6 &8), we performed 2 way analysis of covariance using the python packages statsmodels and pandas, using the output (ppERK) area under the cure as the dependent variable, and intertrial interval and stimulation type (combination or summation) as independent variables. This is explained in the Methods, Simulation and Analysis section. The results for the statistical tests are provided in the captions to Figures 3, 5 and 6, and Figure 8- Supplementary Table 1.

Random forest regression was used to analyze the robustness simulations, to determine which parameter values had the most influence on the change in ppERK AUC versus ITI. This is explained in the Methods, Simulation and Analysis section. The results of the random forest regression are provided in Table 5, and Figure 10.

(For large datasets, or papers with a very large number of statistical tests, you may upload a single table file with tests, Ns, etc., with reference to sections in the manuscript.)

**Group allocation**

- Indicate how samples were allocated into experimental groups (in the case of clinical studies, please specify allocation to treatment method); if randomization was used, please also state if restricted randomization was applied
- Indicate if masking was used during group allocation, data collection and/or data analysis

Please outline where this information can be found within the submission (e.g., sections or figure legends), or explain why this information doesn’t apply to your submission:

Group allocation does not apply because every model was simulated with every condition.

**Additional data files (“source data”)**

- We encourage you to upload relevant additional data files, such as numerical data that are represented as a graph in a figure, or as a summary table
- Where provided, these should be in the most useful format, and they can be uploaded as “Source data” files linked to a main figure or table
- Include model definition files including the full list of parameters used
- Include code used for data analysis (e.g., R, MatLab)
- Avoid stating that data files are “available upon request”

Please indicate the figures or tables for which source data files have been provided:

All model files are freely available on <https://github.com/neurord/ERK/releases/tag/1.0.0>

All programs to analyze simulation output are available on <https://github.com/neurord/NeuroRDanal/releases/tag/2.0.0>.

Programs for the statistical analysis and random forest analysis are available on <https://github.com/neurord/ERK/tree/master/Analysis>.

These URLs are provided in the manuscript methods section. Model files are available from modelDB, accession number 267073.
